# Clinical Predictors of Inpatient Mortality and Poor Postoperative Course After aSAH Microsurgical Clipping: A 10-Year Experience from a Peruvian Tertiary Care Center

**DOI:** 10.3390/jcm14134799

**Published:** 2025-07-07

**Authors:** Fernando Terry, Alejandro Enríquez-Marulanda, Nathaly Chinchihualpa-Paredes, Meiling Carbajal-Galarza, Claudia L Vidal-Cuellar, Guiliana Mas-Ubillus, Bruno Diaz-Llanes, Carlos Quispe-Vicuña, Niels Pacheco-Barrios, Rommel Arbulu-Zuazo, Ziev B. Moses, Joel Sequeiros, Evan Luther, Robert M. Starke, Philipp Taussky, Jaime Lopez-Calle

**Affiliations:** 1 Facultad de Medicina Humana, Universidad de San Martín de Porres, Lima 15101, Peru; fernando_terry@usmp.pe; 2 Department of Neurosurgery, Beth Israel Deaconess Medical Center, Harvard Medical School, Boston, MA 02215, USA; 3 Department of Neurology, University of Cincinnati, Cincinnati, OH 45219, USA; chinchny@ucmail.uc.edu; 4 School of Medicine, Universidad Peruana Cayetano Heredia, Lima 15102, Peru; meiling.carbajal.g@upch.pe (M.C.-G.); claudia.vidalcuellar@qeh.ox.ac.uk (C.L.V.-C.); guiliana.mas.u@upch.pe (G.M.-U.); 5 Department of Neurosurgery, Clínica Internacional, Lima 15001, Peru; brunodiazllanes@gmail.com (B.D.-L.);; 6 Grupo de Investigación Neurociencias, Metabolismo, Efectividad Clínica y Sanitaria (NEMECS), Universidad Científica del Sur, Lima 15142, Peru; vicunas998@gmail.com; 7 Carrera de Medicina Humana, Universidad Científica del Sur, Lima 15142, Peru; 8 Department of Neurosurgery, Hospital Nacional Arzobispo Loayza, Lima 15013, Peru; 9 Department of Neurological Surgery, Division of CNS Endovascular Surgery, University of Louisville, Louisville, KY 40202, USA; 10 Department of Neurosurgery, Allegheny General Hospital, Pittsburgh, PA 15212, USA; 11 Department of Neurological Surgery, University of Miami Miller School of Medicine, Miami, FL 33136, USA

**Keywords:** aneurysmal subarachnoid hemorrhage, ruptured aneurysms, microsurgery, mortality

## Abstract

**Background/Objectives**: Aneurysmal subarachnoid hemorrhage (aSAH) is a medical emergency with a high mortality rate requiring urgent treatment. This study aimed to identify clinical predictors of inpatient mortality and poor postoperative course after aSAH surgical clipping. **Methods**: We performed a retrospective review of medical records for 210 patients with aSAH treated via surgical clipping at our institution between 2010 and 2019. Baseline demographic data and clinical characteristics related to aSAH were collected. To identify factors associated with inpatient mortality and a poor postoperative course after aSAH microsurgical clipping, we conducted a univariate and bivariate analysis, as well as a multivariate analysis via the Poisson regression model. **Results**: The overall cumulative mortality over the 10-year study period was 11.43%. A severe WFNS scale score (aRR: 2.86; 95% CI: 1.28–6.39; *p* = 0.011) and having 1 (aRR: 5.76; 95% CI: 2.02–16.39, *p* = 0.001) or ≥2 (aRR: 18.86; 95% CI: 5.16–68.90, *p* < 0.001) postoperative neurosurgical complications were associated with an increased risk of inpatient mortality. A moderate (aRR: 3.71; 95% CI: 1.45–9.50; *p* = 0.006) or severe (aRR: 4.18; 95% CI: 1.12–15.60; *p* = 0.034) Glasgow scale score on admission, and presenting 1 (aRR: 2.31; 95% CI: 1.27–4.19; *p* = 0.006) or ≥2 postoperative clinical complications (aRR: 3.34; 95% CI: 1.83–6.10; *p* < 0.001) were associated with an increased risk of a poor postoperative course. **Conclusions**: While promising and widely supported by the published literature, these findings require further validation in a larger prospective and multi-centered study to adequately propose health policies on neurointensive care for the Peruvian population. Ultimately, developing socioeconomic setting-focused intervention algorithms and clinical practice guidelines could enhance the survival and postoperative course of patients presenting with aSAH.

## 1. Introduction

Aneurysmatic subarachnoid hemorrhage (aSAH) is a medical emergency accounting for as much as 5% of cerebrovascular events, with a total mortality of 20–35% [1,2]. In addition, it has been associated with negative postoperative clinical outcomes, such as physical disability, functional dependence, and so on, in a third of surviving patients [3,4]. Moreover, given that it mainly affects middle-aged adults, a substantial decrease in work productivity is foreseen [5]. Its worldwide incidence is reported as 7.9 cases per 100,000 people per year [6], varying according to gender, age, and geographical location [7]. From a regional perspective, Latin America shows a lower incidence of aSAH (4.8 cases per 100,000 people per year) compared with other countries, such as Japan and Finland [6]. Nevertheless, further studies are required to determine the current epidemiological trends in low-income countries such as Peru.

The mortality rates for this disease have decreased in recent decades due to the advances made in the understanding of its pathophysiology [8] and the recommendations on its acute treatment [9,10,11]. It is necessary to identify risk factors for poor postoperative outcomes, disability, and mortality to ensure optimal survival outcomes after aSAH. Multiple reviews have reported heavy smoking, arterial hypertension, alcohol consumption, and non-white race as risk factors [12,13]. Nevertheless, most studies have been conducted in high-income countries, with a notable lack of research in middle- and low-income settings [8]. Surgical treatment of SAH is based on patient stabilization, the prevention of rebleeding, and the management of neurological complications. One of the mainstays is the exclusion of the ruptured aneurysm via surgical clipping or endovascular embolization, with the aim of preventing rebleeding. Additionally, it requires the control of intracranial pressure, the management of cerebral vasospasm, and the maintenance of adequate cerebral perfusion. The choice of surgical approach depends on factors such as the patient’s clinical condition, the aneurysm’s location and morphology, and the treating team’s experience [14].

Low- and middle-income countries face significant challenges in accessing and delivering adequate surgical care, which results in higher mortality and disability rates [15]. Peru, a country with vast socioeconomic and geographic diversity, exemplifies these challenges [16]. The treatment of aSAH requires early and effective intervention to improve the patient’s prognosis. However, achieving an early surgical intervention depends on a rapid medical response, access to diagnostic tools such as tomography and angiography, and the availability of skilled surgical teams [17]. Unfortunately, many healthcare facilities in Peru lack the necessary infrastructure, which leads to delays in diagnosis, referral, and treatment [18]. Given the stark differences between the healthcare realities in high-income countries, where most studies have been conducted, it is vital to identify the unique risk factors for poor outcomes [16].

Ruptured aneurysms can be managed via endovascular embolization or surgical clipping [19]. In most cases, the aneurysm’s shape, parent vessel anatomy, and location determine the preferred treatment approach. At present, microsurgical clipping is the preferred treatment for ruptured middle cerebral artery (MCA) aneurysms [20,21,22]. The literature reports the time of surgery, history of hypertension, smoking, advanced age, and a high World Federation of Neurosurgical Societies (WFNS) score, among others [23,24,25,26], as predictors of the postoperative course; nonetheless, as study designs and eligibility criteria tend to be highly variable, it is important to interpret them according to the clinical scenario to ensure their rapid and appropriate management. An aSAH mortality rate of 27.5% has been reported in the Peruvian context [27]; however, there is a marked limitation due to the small number of studies published, impairing the proper identification of predictors for a poor postoperative course. 

Given this, our study aimed to identify clinical predictors of inpatient mortality and a poor postoperative course after surgical clipping for aSAH in a Peruvian tertiary care center between 2010 and 2019.

## 2. Materials and Methods

### 2.1. Study Cohort and Patient Selection

This retrospective analysis utilized a prospectively collected database from a single tertiary care center in Peru (“Hospital Nacional Arzobispo Loayza (HNAL)”), including all the adult patients with an angiographic or angio-tomographic diagnosis of aSAH and treated via microsurgical clipping between 2010 and 2019. Patients were identified from institutional medical records using the ICD-10 code 60.0. The exclusion criteria included sentinel bleeding from unruptured aneurysms, death before hospital admission, and a lack of follow-up data. This study was approved by the local Institutional Review Board (IRB) with protocol #02687-2022, and informed consent was waived due to the observational nature of the study. This study adhered to the Strengthening Reporting of Observational Studies in Epidemiology (STROBE) reporting guidelines (Supplementary Table S1) [28].

### 2.2. Demographic and Clinical Characteristics

Data were collected using a standardized data sheet, including the following characteristics: patient demographics [gender (male or female), age (in years), comorbidities (none; ≥1), and family history of aneurysms]; clinical characteristics [thunderclap headache, time (in days) from aSAH onset to admission, Glasgow scale score on admission (mild [13–15 points], moderate [9–12 points], or severe [<9 points]), WFNS scale score on admission (mild [1–3 points] or severe [4–5 points]), diagnostic test (computed tomography angiography [CTA], angiography, or both), Fisher computerized tomography (CT) scale score on admission (class I, II, III, or IV), and aneurysms per patient]; aneurysm characteristics [location, maximum diameter (small/medium [7–14 mm], large [15–25 mm], or giant [>25 mm])]; and intervention characteristics [craniotomy approach (mini-pterional, lateral supraorbital, frontal, or decompressive hemicraniectomy), number of clipped aneurysms, need for proximal control with transient parent artery clipping, and transient clipping time].

### 2.3. Postoperative Outcomes

The primary outcome was the overall inpatient mortality, defined as death from any cause occurring after discharge from the ICU and during hospitalization in the neurosurgery service. The secondary outcome was a poor postoperative course, measured with the modified Rankin scale (mRS) score at discharge (no or mild disability [0–2 points] or disability [3–6 points]).

### 2.4. Statistical Methods

Categorical variables are presented as frequencies and percentages. Continuous variables are presented as medians (interquartile ranges [IQRs] or means ± standard deviations (SDs), depending on the normality of the data, assessed using the Shapiro–Wilk test. The bivariate analysis included the chi-square test or Fisher’s exact test to analyze the categorical variables, and an unpaired Student’s t-test or the Mann–Whitney U test, depending on the normality of continuous data, in order to compare the characteristics between the cohorts according to the outcome distribution.

For the multivariate analysis, clinically significant variables identified in the univariate and bivariate analyses were included in a Poisson regression with a robust variance estimator [29]. Both unadjusted and adjusted Poisson regression models were built to identify significant clinical predictors of inpatient mortality and a poor postoperative course (the mRS score at discharge). The crude (cRR) and adjusted (aRR) relative risks, 95% confidence intervals (CIs), and *p*-values are reported. Statistical significance was set at *p* < 0.05. All statistical analyses were performed using STATA 18.0/BE. 

## 3. Results

### 3.1. Baseline Characteristics 

**Figure 1:**
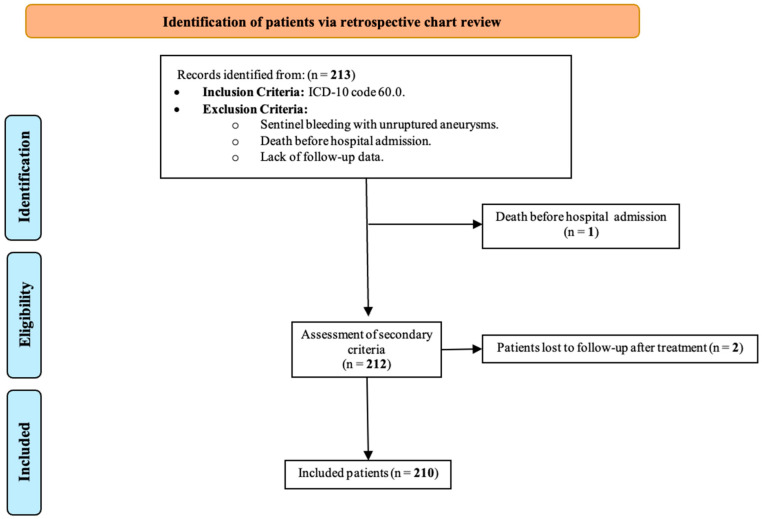
STROBE patient selection flowchart.

Of the 213 patients initially identified via the retrospective screening of medical records, only 3 were excluded (Figure 1). A total of 210 patients were included in the final analysis. The male population was predominant (144/210 (68.6%)), with a median age of 52.5 years. The highest proportion of participants were ≤ 65 years old (178/210 (84.8%)), with at least one comorbidity (87/210 (52.7%)) and no family history of aneurysms (162/210 (98.8%)). Most cases presented as a thunderclap headache (207/210 (99.0%)), with a mild Glasgow scale score (181/210 (86.6%)) and a mild WFNS scale score (184/210 (87.6%)). The median time from aSAH presentation to admission was 2 days, and most patients received surgical intervention more than 10 days after aSAH presentation (92/210 (45.3%)). The most common diagnostic method was CTA (168/210 (80.4%)). The most frequent Fisher CT classification was class III (121/210 (57.9%)), and there was a median of one aneurysm per patient (Table 1). A total of 15 patients presented with rebleeding during the waiting time. Other clinical characteristics and perioperative complications are described in Tables S2 and S3.

**Figure 2:**
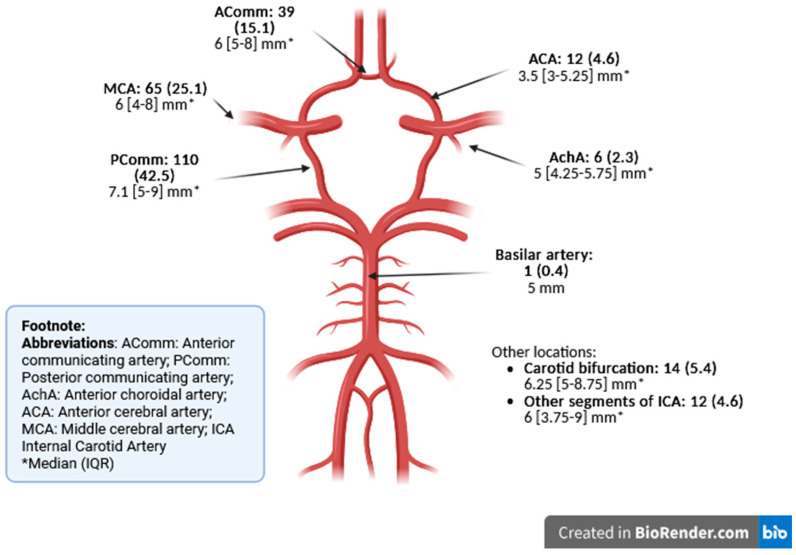
Maximum aneurysm diameters according to location (n = 259).

Regarding the aneurysm characteristics according to location, a total of 259 aneurysms were identified among the 210 included patients. A single aneurysm was identified in 171 patients, 2 in 32 patients, 3 in 4 patients, and 4 in 3 patients. Most aneurysms occurred in the anterior circulation (258/259 (99.6%)), with the highest frequency emerging from the posterior communicating artery (PComm) (110/259 (42.5%)) (Figure 2). Most of the aneurysms were classified as having a small/medium maximum diameter (253/259 (97.7%)), and only 6 aneurysms were deemed as large (Table 2).

Regarding the intervention characteristics, most had a mini-pterional approach (184/210 (87.2%)), single-aneurysm clipping (171/210 (81.4%)), and no need for transient clipping in the parent artery (155/259 (59.8%)). For both the first and second aneurysms, the time of transient clipping mostly lasted for 5–10 min (45/102 (44.12%) and 10/22 (45.45%), respectively) (Table 3). External ventricular drainage (EVD) was performed before surgery in only four cases of acute symptomatic hydrocephalus. Meanwhile, microsurgical fenestration of the lamina terminalis was performed before clipping in those cases with asymptomatic hydrocephalus or persistent cerebral edema despite cisternal opening (7.14%). The patients’ pre-, intra-, and postoperative characteristics are described in Table S4.

### 3.2. Patient Characteristics According to Mortality and Postoperative Outcomes

A total of 24 (11.4%) patients were deceased after microsurgical clipping, with a higher proportion among those with a severe Glasgow scale score on admission (7/10 (70.0%); *p* < 0.001), a severe WFNS scale score on admission (9/26 (34.6%); *p* < 0.001), at least one neurosurgical intraoperative complication (10/87 (18.3%); *p* = 0.022), at least two neurosurgical postoperative complications (7/13 (53.8%); *p* < 0.001), other neurosurgical postoperative complications (5/10 (50.0%); *p* < 0.001), and a clinical postoperative complication (9/45 (20.0%); *p* = 0.026) (Table 4 and Table S5).

Less than half (56/210 (26.7%)) of the population had moderate/severe disability at discharge in activities of daily living after the aneurysm microsurgical clipping, with a higher proportion among those older than 65 years on admission (14/32 (43.7%); *p* = 0.018), a severe Glasgow scale score on admission (10/10 (100.0%); *p* = 0.009), a severe WFNS clinical scale score on admission (17/26 (65.4%); *p* < 0.001), a need for transient clipping of the parent artery (34/103 (33.0%); *p* = 0.041), ≥1 intraoperative neurosurgical complication (23/65 (35.4%); *p* = 0.049), ≥2 postoperative neurosurgical complications (10/13 (76.9%); *p* < 0.001), ≥1 cerebral infarction and/or vasospasm (22/45 (48.9%); *p* < 0.001), and ≥2 postoperative clinical complications (18/31 (58.1%); *p* < 0.001) (Table 4).

At 6 months, only 25 patients reported mRS data, with the majority (12/25 (48%)) reporting mild disability (score: 1). At the 1-year follow-up, only 19 patients reported mRS data, with the majority (12/19 (63.2%)) also reporting mild disability (Table S4).

Around 14.97% (28/187) of the population presented a moderate/severe disability at discharge, with a higher proportion among those older than 65 years (8/27 (29.6%); *p* = 0.021), with a severe Glasgow scale score on admission (100.0%; *p* < 0.001), with a severe WFNS clinical scale score on admission (7/17 (41.2%); *p* = 0.001), with ≥2 postoperative neurosurgical complications (2/6 (33.3%); *p* = 0.033), with other postoperative neurosurgical complications (3/6 (50.0%); *p* = 0.018), and ≥2 postoperative clinical complications (9/25 (36.0%); *p* = 0.001) (Table S5).

### 3.3. Clinical Predictors of Inpatient Mortality

A first Poisson regression model was constructed to identify clinical predictors for inpatient mortality. The unadjusted model revealed the following predictors of mortality: a severe Glasgow score on admission (RR: 8.45; 95% CI: 4.48–15.92; *p* < 0.001), a severe WFNS scale score on admission (RR: 4.25; 95% CI: 2.07–8.71; *p* < 0.001), ≥1 intraoperative neurosurgical complications (RR: 2.4; 95% CI: 1.12–5.16; *p* = 0.025), ≥2 postoperative neurosurgical complications (RR: 11.77; 95% CI: 4.86–28.48; *p* < 0.001), ≥1 cerebral infarction and/or vasospasm (RR: 5.83; 95% CI: 2.43–13.95; *p* < 0.001), and ≥2 postoperative clinical complications (RR: 2.73; 95% CI: 1.05–7.12; *p* = 0.040).

After adjusting for the baseline characteristics, one postoperative neurosurgical complication (aRR: 5.76; 95% CI: 2.02–16.39; *p* = 0.001), ≥2 complications (aRR: 18.86; 95% CI: 5.16–68.90; *p* < 0.001), and a severe WFNS scale score on admission (aRR: 2.86; 95% CI: 1.28–6.39; *p* = 0.011) remained as strong predictors of inpatient mortality (Table 5).

### 3.4. Clinical Predictors of Poor Postoperative Course 

A second Poisson regression model was constructed to identify clinical predictors of a poor postoperative course (with disability defined as an mRS score of 3–6). The unadjusted model revealed the significance of all the variables assessed. The adjusted model identified that a moderate (aRR: 3.71; 95% CI: 1.45–9.50; *p* = 0.006) or severe (aRR: 4.18; 95% CI: 1.12–15.60; *p* = 0.034) Glasgow scale score on admission, presenting cerebral infarction and/or vasospasm (aRR: 2.25; 95% CI: 1.39–3.66; *p* = 0.001) or other postoperative neurosurgical complications (aRR: 3.11; 95% CI: 1.40–6.91; *p* = 0.005), and presenting one (aRR: 2.31; 95% CI: 1.27–4.19; *p* = 0.006) or ≥2 postoperative clinical complications (aRR: 3.34; 95% CI: 1.83–6.10; *p* < 0.001) were associated with an increased risk of having functional disability in daily activities at discharge (Table 6).

Another Poisson regression model identified a moderate (aRR: 14.64; 95% CI: 4.28–50.09; *p* < 0.001) or severe (aRR: 60.07; 95% CI: 11.96–301.62; *p* < 0.001) Glasgow scale score on admission, other neurosurgical complications (aRR: 8.29; 95% CI: 2.60–26.44; *p* < 0.001), and ≥2 postoperative clinical complications (aRR: 4.04; 95% CI: 1.67–9.79; *p* = 0.002) as significant predictors of a moderate/severe Glasgow scale score at discharge. A severe WFNS scale on admission (aRR: 0.15; 95% CI: 0.04–0.54; *p* = 0.004) was associated with a lower risk of a moderate/severe Glasgow scale score at discharge (Table S6).

**Figure 3:**
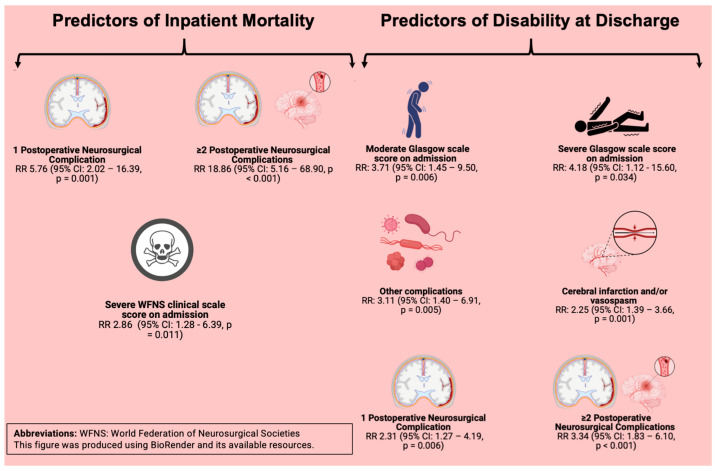
Predictors of Inpatient Mortality and Disability at Discharge.

A summary of the clinical predictors is presented in Figure 3. 

### 3.5. Sensitivity Analysis

Considering that the occurrence of aSAH is age-dependent, we analyzed the clinical predictors according to age. For patients aged up to 65 years, only those with cerebral infarction and/or vasospasm and those with other postoperative neurosurgical complications had significantly higher mortality. Furthermore, this same pattern was repeated only for moderate/severe disability. We also added the variable of ≥2 postoperative clinical complications as a significant predictor. The variable of intraoperative neurosurgical complications was excluded to emphasize the impact of both clinical and neurosurgical postoperative complications. We found that a severe WFNS score on admission or presenting any postoperative clinical or neurosurgical complication significantly increased the risk of moderate/severe disability (Tables S7 and S8).

## 4. Discussion

This decade-long retrospective study provides valuable information on the clinical predictors of in-hospital mortality and a poor postoperative course in patients with aneurysmal SAH after microsurgical clipping at a Latin American high-volume center. Our findings reveal that a severe WFNS score and a moderate/severe Glasgow scale score on admission, as well as presenting postoperative neurosurgical or clinical complications, were associated with an increased risk of mortality and poor postoperative course. These results align with those observed in high-income settings, despite the unique challenges faced within the Peruvian healthcare system.

An overall mortality of 11.43% was observed among patients undergoing surgical clipping, which is consistent with the results of other observational studies. Deutsch et al. (2018) [30] and Ikawa et al. (2020) [31] reported in-hospital mortality rates after aneurysm surgical clipping of 11.4% and 7.1%, respectively. Over the past 40 years, the SAH case fatality rate decreased at a worldwide level by an average of −1.5%/year [32]. In addition, presenting a severe WFNS clinical scale score and postoperative neurosurgical complications significantly increased the risk of mortality, similar to those findings from high-income countries. For instance, Odensass et al. (2024) [33] found that the risk was almost doubled (HR: 1.53; 95% CI: 1.06–2.22; *p* = 0.025), and Said et al. (2024) [34] found that a WFNS score of >3 (OR: 3.87; 95% CI: 2.83–5.28; *p* < 0.001) was strongly associated with an increased risk of mortality. This is because poor clinical conditions (WFNS > 3) and severe SAH (Fisher classes III-IV) on admission were established as risk factors for death according to the published literature [23], while a high WFNS score (4–5) was more strongly associated with an increased risk of a poor postoperative course at 90 days (aOR: 6.38; 95% CI: 2.66–15.31; *p* < 0.001) in patients with aSAH [35].

A moderate/severe Glasgow scale score on admission and postoperative complications were significantly associated with an increased risk of postoperative disability. Turek et al. (2016) [36] evaluated 190 Polish patients and found that postoperative complications were associated with an increased risk of a poor postoperative course (OR: 15.4; 95% CI: 3.3–73.0; *p* < 0.001). Bae et al. (2021) [37] evaluated the predictive ability of a modified scale in patients with aSAH by combining the GCS with the modified Fisher scale (GCS-F). The study found that lower scores for the GCS-F at admission were associated with less-favorable outcomes (OR: 0.53; 95% CI: 0.33–0.85; *p* = 0.0094) [37].

A notable finding of our study was that a severe WFNS score on admission and the occurrence of cerebral infarcts were associated with a lower risk of moderate/severe Glasgow scale scores at discharge. This could be due to a paradoxical effect where patients with these factors—thus classified as more-severe cases—receive more intensive and prioritized care during the postoperative period; a scenario that has also been reported in international cohorts of SAH patients [38], although they underwent endovascular treatment. However, this result might be biased by our small sample size, so this finding requires further investigation to fully understand the underlying mechanisms, especially in low–middle-income settings such as Peru, where disparities in healthcare may influence clinical outcomes [39].

Peru faces one of the highest levels of inequality in the world, with significant socioeconomic and geographical disparities that affect healthcare delivery [16,40]. In 2019, Peru allocated just USD 370 per person to healthcare, compared with USD 10,866 per person in the United States and between USD 4500 and USD 6500 in countries such as the United Kingdom, Australia, and Germany. These limited financial resources contribute to a healthcare environment that is both financially constrained and systemically inequitable. Notably, this is reflected in limitations in healthcare delivery (especially to rural regions) and a severe shortage of healthcare professionals (with only 0.82 physicians per 1000 people, most of whom are concentrated in the capital city, Lima) and trained personnel in general [16]. Moreover, the lack of adequate infrastructure, essential medicines, and logistical resources further exacerbates inequalities within the Ministry of Health.

Although tertiary-level hospitals in Peru provide more specialized care [41], most healthcare facilities lack the capacity for urgent care, and those with neuroimaging capabilities are typically centralized or only available in the private sector [18]. These systemic barriers worsen patient severity upon admission and significantly increase the likelihood of postoperative complications. In many cases, the absence of specialized diagnostic tools and the unavailability of skilled medical professionals delay timely intervention, contributing to adverse outcomes.

We found that most patients received surgical treatment more than 10 days after the presentation of aSAH. This is alarming, considering delays in treatment beyond 12 hours worsen patients’ overall prognosis [38], explaining the mortality and poor postoperative course reported in our study. This delay may be due to several causes, such as diagnostic errors, which may occur in up to 14% of cases of acute SAH [42], and up to 70% of delays may stem from the same cause [43]. Similar rates have been reported in Lima, where one study reported an average time from symptom onset to surgical intervention of 7.1 days [17]. Another study reported that more than half of the patients were operated on within the first 96 h after symptom onset; however, there were still cases with significant delays [44]. Another cause of these delays can be attributed to limitations in hospital infrastructure, the availability of specialized resources, a weak referral system between health centers within the country, and delayed clinical diagnosis due to a lack of professional expertise [18].

Our results reinforce the existing evidence on determinants of aSAH patient prognosis. This highlights the need for a thorough initial evaluation and perioperative strategies encompassed in health policies aimed at minimizing complications, aiming to improve functional outcomes and reduce postoperative disability.

We must outline the continued preference for microsurgical clipping over endovascular techniques in many developing countries, such as Peru. Other studies conducted in Peru have also reported this preference. For instance, a study carried out in a public referral hospital revealed that all patients were treated with surgical clipping [17], while in a private hospital, 65.2% of patients received clipping treatment [45]. While endovascular coiling has become the standard approach in high-income countries due to its less invasive nature and shorter recovery times, it remains less commonly used in many low–middle-income settings [46,47]. Additionally, clipping offers the advantage of achieving complete occlusion of the aneurysm, preventing recurrence, and not requiring prolonged anticoagulation therapy [48]. Furthermore, the lack of infrastructure and materials for embolization, especially in the public sector, limits access to the endovascular approach [45]. A cost-effectiveness study conducted in Colombia, a middle-income country, found that clipping is the most cost-effective option for treating anterior circulation aneurysms [49]. This makes surgical clipping a more viable option in settings where healthcare resources are limited, particularly in the public healthcare system.

Another aspect to be addressed is the presence of hydrocephalus and vasospasm, which are two relevant complications related to aSAH. Our study reported that 4 patients (1.9%) presented with hydrocephalus, 28 (13.3%) presented with vasospasm, and only 1 presented with both complications. These rates are lower than those reported in the published literature, where hydrocephalus and vasospasm have shown a prevalence of 15% [50] and 38.4% [51], respectively. These lower rates could stem from the small sample size addressed, supporting the need to evaluate these complications in future studies. 

This study had several limitations. First, the retrospective design introduced the potential for recall bias and may have excluded patients with incomplete data or those lost to follow-up. Moreover, it restricted the integrity and reliability of the data by favoring the presence of referral and selection bias. Second, the prioritized outcomes were only collected at discharge, and no long-term follow-up was performed, limiting the further identification of risk factors and underestimating the significance of those already identified. Third, the single-center design limited the generalizability of our findings, as the population was largely homogeneous, representing a low-to-middle socioeconomic status with preferred treatment in a public tertiary care center. Fourth, the relatively small sample size (n = 210) limited the statistical power of our analysis. Lastly, there is the risk of not having considered other confounding variables that influence the likelihood of inpatient mortality or poor postoperative course. Despite these limitations, our study holds significant value for understanding the Peruvian context. The “Hospital Nacional Arzobispo Loayza (HNAL),” being a high-complexity tertiary center in Lima, provides a representative overview of patients with SAH treated at advanced healthcare facilities within the country. Our findings reflect the challenges and constraints within the Peruvian healthcare system, including the limited availability of specialized resources, the centralization of neuroimaging services, and causes for delay in treatment. In addition, various statistical models were used to ensure adequate data analysis and control for potential confounders.

## 5. Conclusions

We observed an overall mortality and postoperative functional disability of 11.43% and 26.6%, respectively. These outcomes were conditioned by predictors such as a severe WFNS score on admission, a moderate/severe Glasgow scale score on admission, and presenting postoperative neurosurgical complications. While promising and widely supported by the published literature, these findings require further validation in a larger prospective and multi-centered study to adequately propose health policies on neurointensive care for the Peruvian population. Ultimately, developing socioeconomic setting-focused intervention algorithms and clinical practice guidelines could enhance the survival and postoperative course of patients presenting with aSAH. 

## Figures and Tables

**Table 1:** Baseline characteristics (n = 210).

Baseline Characteristics		
Gender, n (%)	Female	66 (31.4)
	Male	144 (68.6)
Age median (IQR), years	52.5 (22)	
Age group, n (%)	≤65 yo	178 (84.8)
	>65 yo	32 (15.2)
Comorbidities, n (%)	None	78 (47.3)
	≥1	87 (52.7)
Familiar aneurysm history, n (%)	No	162 (98.8)
	Yes	2 (1.2)
Thunderclap headache	No	2 (1.0)
	Yes	207 (99.0)
aSAH-to-admission median (IQR), days	2 (6.42)	
Glasgow scale on admission	Mild (13–15)	181 (86.6)
	Moderate (9–12)	18 (8.6)
	Severe (<9)	10 (4.8)
WFNS SAH scale on admission	Mild (1–3)	184 (87.6)
	Severe (4–5)	26 (12.4)
aSAH-to-surgery, median (IQR), days	10 (13)	
aSAH-to-surgery, median (IQR), days	≤3 days	43 (21.2)
	4–10 days	68 (33.5)
	>10 days	92 (45.3)
Diagnostic test, n (%)	CT angiography	168 (80.4)
	Angiography	18 (8.6)
	Both	23 (11.0)
Fisher CT scale on admission	Class I	9 (4.3)
	Class II	20 (9.6)
	Class III	121 (57.9)
	Class IV	59 (28.2)
Aneurysms per patient, median (IQR)	1 (0)	

Categorical variables are described using frequencies and proportions. Continuous variables are described using medians and interquartile ranges. Abbreviations: WFNS: World Federation of Neurological Surgeons; aSAH: aneurysmatic subarachnoid hemorrhage; IQR: interquartile range; CT: computed tomography.

**Table 2:** Aneurysm characteristics according to location (n = 259).

Characteristics		Aneurysm Location (n = 259)							
		Anterior Circulation (n = 258)							Posterior Circulation (n = 1)
		AComm (n = 39)	ACA (n = 12)	MCA (n = 65)	AchA (n = 6)	Carotid Bifurcation (n = 14)	ICA (n = 12)	PComm (n = 110)	Basilar Artery (n = 1)
Maximum diameter	Small/medium (<15 mm)	39 (100) 6 [5,6,7,8] *	12 (100) 3.5 [3–5.25] *	63 (96.9) 6 [4,5,6,7,8] *	6 (100) 5 [4.25–5.75] *	13 (92.9) 6 [5,6,7,8] *	11 (91.7) 6 [3.5–8.5] *	108 (98.2) 7 [5,6,7,8,9] *	1 (100) 5 [5] *
	Large (15–25 mm)	-	-	2 (3.1) 18 [16,17,18,19,20] *	-	1 (7.1) 20 [20] *	1 (8.3) 16 [16] *	2 (1.8) 17.5 [15,16,17,18,19,20] *	-
	Giant (>25 mm)	-	-	-	-	-	-	-	-
Microsurgical clipping		38 (16.4)	12 (5.2)	55 (23.7)	6 (2.6)	12 (5.2)	8 (3.5)	101 (43.5)	-

* Median [IQR] in mm. Abbreviations: AComm: anterior communicating artery; PComm: posterior communicating artery; AchA: anterior choroidal artery; ACA: anterior cerebral artery; MCA: middle cerebral artery; ICA: internal carotid artery.

**Table 3:** Intervention characteristics.

Clipping Characteristics	
Craniotomy approach, n (%)	
Mini-pterional	184 (87.2)
Lateral supraorbital	10 (4.7)
Frontal	10 (4.7)
Decompressive hemicraniectomy	7 (3.3)
Transitory clipping in parenteral artery	104 (40.2)
Transitory clipping time in 1st aneurysm	
<5 min	30 (29.41)
5–10 min	45 (44.12)
>10 min (prolonged)	27 (26.47)
Transitory clipping time in 2nd aneurysm	
<5 min	8 (36.36)
5–10 min	10 (45.45)
>10 min (prolonged)	4 (18.18)
Transitory clipping time in 3rd aneurysm	
<5 min	4 (100)
5–10 min	0
>10 min (prolonged)	0
Transitory clipping time in 4th aneurysm	
<5 min	1 (100)
5–10 min	0
>10 min (prolonged)	0

Categorical variables are described using frequencies and proportions. Continuous variables are described using medians and interquartile ranges.

**Table 4:** Patient characteristics according to mortality and mRS at discharge.

Characteristics		Mortality		*p*	mRS		*p*
		No (n = 186), n (%)	Yes (n = 24), n (%)		No/Mild Disability (n = 154), n (%)	Moderate/Severe Disability (n = 56), n (%)	
Gender				0.800 ^a^			0.638 ^a^
	Female	59 (89.4)	7 (10.6)		47 (71.2)	19 (28.8)	
	Male	127 (88.2)	17 (11.8)		107 (74.3)	37 (25.7)	
Age				0.418 ^a^			**0.018 ^a^**
	≤65 years old	159 (89.3)	19 (10.7)		136 (76.4)	42 (23.6)	
	>65 years old	27 (84.4)	5 (15.6)		18 (56.3)	14 (43.7)	
History of any chronic disease				0.993 ^a^			0.426 ^a^
	None	69 (88.5)	9 (11.5)		59 (75.6)	19 (24.4)	
	≥1	77 (88.5)	10 (11.5)		61 (70.1)	26 (29.9)	
Time from stroke to admission (in days), median (IQR)		2 (6.5)	5.5 (7.0)	0.060 ^b^	2 (6.5)	2.5 (6)	0.743 ^b^
Glasgow scale score on admission, categorized				**<0.001 ^a^**			**0.009 ^a^**
	Mild (15–13)	166 (91.7)	15 (8.3)		144 (79.6)	37 (20.4)	
	Moderate (12–9)	17 (94.4)	1 (5.6)		10 (55.6)	8 (44.4)	
	Severe (<9)	3 (30.0)	7 (70.0)		0 (0.0)	10 (100.0)	
WFNS clinical scale score on admission				**<0.001 ^a^**			**<0.001 ^a^**
	Mild (1 to 3)	169 (91.8)	15 (8.1)		145 (78.8)	39 (21.2)	
	Severe (4 to 5)	17 (65.4)	9 (34.6)		9 (34.6)	17 (65.4)	
Fisher tomographic scale score on admission				0.516 ^a^			0.260 ^a^
	I—no evidence of bleeding in cisterns or ventricles.	9 (100.0)	0 (0.0)		9 (100.0)	0 (0.0)	
	II—thin diffuse blood, with a layer of < 1 mm in cisterns measured vertically	19 (95.0)	1 (5.0)		16 (80.0)	4 (20.0)	
	III—thick cisternal clot of >1 mm in cisterns, measured vertically	107 (88.4)	14 (11.6)		87 (71.9)	34 (28.1)	
	IV—intraparenchymal hematoma, intraventricular hemorrhage, and +/− diffuse bleeding	51 (86.4)	8 (13.6)		42 (71.2)	17 (28.8)	
Time from stroke to operation (in days), median (IQR)		9 (12.0)	10 (23.0)	0.480 ^b^	9 (12.0)	10 (12.0)	0.708 ^b^
Need for transient clipping in parenteral artery				0.161 ^a^			**0.041 ^a^**
	No	98 (91.6)	9 (8.4)		85 (79.4)	22 (20.6)	
	Yes	88 (85.4)	15 (14.6)		69 (77.0)	34 (33.0)	
Intraoperative neurosurgical complications				**0.022 ^a^**			**0.049 ^a^**
	None	132 (92.3)	11 (7.7)		111 (77.6)	32 (22.4)	
	≥1	49 (81.7)	11 (18.3)		42 (64.6)	23 (35.4)	
Postoperative neurosurgical complications				**<0.001 ^a^**			**<0.001 ^a^**
	None	146 (95.4)	7 (4.6)		128 (83.7)	25 (16.3)	
	1	32 (76.2)	10 (23.8)		22 (52.4)	20 (47.6)	
	≥2	6 (46.1)	7 (53.8)		3 (23.1)	10 (76.9)	
Specific postoperative neurosurgical complications				**<0.001 ^a^**			**<0.001 ^a^**
	No complications	146 (95.4)	7 (4.6)		128 (52.0)	25 (47.9)	
	≥1 cerebral infarction and/or vasospasm	33 (73.3)	12 (26.7)		23 (51.1)	22 (48.9)	
	Other complications	5 (50.0)	5 (50.0)		2 (20.0)	8 (80.0)	
Postoperative clinical complications				**0.026 ^a^**			**<0.001 ^a^**
	None	118 (92.9)	9 (7.1)		111 (87.4)	16 (12.6)	
	1	36 (80.0)	9 (20.0)		25 (55.6)	20 (44.4)	
	≥2	25 (80.6)	6 (19.3)		13 (41.9)	18 (58.1)	

Categorical variables are described using frequencies and proportions. Continuous variables are described using medians and interquartile ranges. Statistical tests: ^a^ Pearson’s chi-squared test; ^b^ Mann–Whitney U test. *p*-values < 0.05 are in bold. **Abbreviations:** mRS: modified Rankin functional scale; WFNS: World Federation of Neurosurgical Societies.

**Table 5:** Poisson regression analysis predicting inpatient mortality.

Variable		Crude Model			Adjusted Model *		
		RR	95% CI	*p*	RR	95% CI	*p*
Age							
	≤65 years old	Ref.			Ref.		
	>65 years old	1.46	0.59–3.65	0.413	0.68	0.27–1.71	0.410
Glasgow scale score on admission, categorized							
	Mild (15–13)	Ref.			Ref.		
	Moderate (12–9)	0.67	0.09–4.81	0.691	-	-	-
	Severe (<9)	8.45	4.48–15.92	**<0.001**	-	-	-
WFNS clinical scale score on admission							
	Mild (1 to 3)	Ref.			Ref.		
	Severe (4 to 5)	4.25	2.07–8.71	**<0.001**	2.86	1.28–6.39	**0.011**
Intraoperative neurosurgical complications							
	None	Ref.			Ref.		
	≥1	2.4	1.12–5.16	**0.025**	1.40	0.63–3.40	0.404
Postoperative neurosurgical complications							
	None	Ref.			Ref.		
	1	5.2	2.1–12.87	**<0.001**	5.76	2.02–16.39	**0.001**
	≥2	11.77	4.86–28.48	**<0.001**	18.86	5.16–68.90	**<0.001**
Specific postoperative neurosurgical complications							
	No complications	Ref.			Ref.		
	At least cerebral infarction and/or vasospasm	5.83	2.43–13.95	**<0.001**	0.47	0.20–1.11	0.086
	Other complications	10.93	4.20–28.40	**<0.001**	-	-	-
Reported postoperative clinical complications							
	None	Ref.			Ref.		
	1	2.82	1.19–6.68	**0.018**	1.90	0.80–4.53	0.146
	≥2	2.73	1.05–7.12	**0.040**	2.23	0.79–6.31	0.131

Unadjusted and adjusted Poisson regression model results are reported as RRs (95% CI and *p*-value). **Abbreviations:** RR: risk ratio; CI: confidence interval; WFNS: World Federation of Neurosurgical Societies. **Statistical test**: Generalized linear model [family(Poisson) link(log)] (robust). *p*-values < 0.05 are in bold. * Adjusted for all variables from the table except Glasgow score on admission, categorized.

**Table 6:** Poisson regression analysis predicting mRS at discharge.

Variable		mRS (Disability)						
		Crude Model				Adjusted Model **		
		RR	95% CI		*p*	RR	95% CI	*p*
Age								
	≤65 years old	Ref.				Ref.		
	>65 years old	1.85	1.15–2.98		**0.011**	1.26	0.72–2.20	0.423
Glasgow scale score on admission, categorized								
	Mild (15–13)	Ref.				Ref.		
	Moderate (12–9)	2.17	1.20–3.93		**0.010**	3.71	1.45–9.50	**0.006**
	Severe (<9)	4.89	3.67–6.53		**<0.001**	4.18	1.12–15.60	**0.034**
WFNS clinical scale score on admission								
	Mild (1 to 3)	Ref.				Ref.		
	Severe (4 to 5)	3.08	2.08–4.58		**<0.001**	0.39	0.11–1.35	0.137
Need for transient clipping in parenteral artery								
	No	Ref.						
	Yes	1.61	1.01–2.55		**0.046**	1.44	0.85–2.41	0.173
Intraoperative neurosurgical complications								
	None	Ref.				Ref.		
	≥1	1.58	1.01–2.48		**0.046**	-	-	-
Postoperative neurosurgical complications								
	None	Ref.				Ref.		
	1	1.49	0.93–2.39		0.098	1.06	0.61–1.82	0.844
	≥2	2.68	1.23–5.85		**0.013**	1.50	0.58–3.90	0.405
Specific postoperative neurosurgical complications								
	No complications	Ref.				Ref.		
	Cerebral infarction and/or vasospasm	2.99	1.87–4.78		**<0.001**	2.25	1.39–3.66	**0.001**
	Other complications	4.90	3.04–7.87		**<0.001**	3.11	1.40–6.91	**0.005**
Postoperative clinical complications								
	None		Ref.				Ref.	
	1	3.53	2.01–6.20		**<0.001**	2.31	1.27–4.19	**0.006**
	≥2	4.61	2.66–7.98		**<0.001**	3.34	1.83–6.10	**<0.001**

Unadjusted and adjusted Poisson regression models are reported as RRs (95% CI and *p*-value). **Abbreviations:** RR: risk ratio; CI: confidence interval; WFNS: World Federation of Neurosurgical Societies. **Statistical test:** Generalized linear model [family(Poisson) link(log)] (robust). *p*-values < 0.05 are in bold. ** Adjusted for all variables from the table.

## Data Availability

Additional information about the statistical analysis and propensity score matching can be found in the Supplementary Materials.

## Supplementary Materials

The following supporting information can be downloaded at https://www.mdpi.com/article/10.3390/jcm14134799/s1. Table S1. STROBE statement: checklist of items that should be included in reports of cohort studies. Table S2. Clinical characteristics of patients operated on for aneurysmal subarachnoid hemorrhage seen in the neurosurgery service of the Hospital Nacional Arzobispo Loayza during 2010–2019. Table S3. Perioperative complications of patients operated on for aneurysmal subarachnoid hemorrhage at the neurosurgery service of the Hospital Nacional Arzobispo Loayza during 2010–2019. Table S4. Discharge and follow-up status of patients operated on for aneurysmal subarachnoid hemorrhage at the neurosurgery service of the Hospital Nacional Arzobispo Loayza during 2010–2019. Table S5. Characteristics of patients operated on for aneurysmal subarachnoid hemorrhage in the neurosurgery service of the Hospital Nacional Arzobispo Loayza during 2010–2019, according to Glasgow Scale at discharge. Table S6. Poisson regression analysis predicting moderate/severe Glasgow Scale scores at discharge. Table S7. Sensitivity analysis for mortality risk in patients operated on for aneurysmal subarachnoid hemorrhage at the neurosurgery service of the Hospital Nacional Arzobispo Loayza during 2010–2019. Table S8. Sensitivity analysis for mRS (disability) risk in patients operated on for aneurysmal subarachnoid hemorrhage at the neurosurgery service of the Hospital Nacional Arzobispo Loayza during 2010–2019.

## Abbreviations

The following abbreviations were used in this manuscript:

aSAH	Aneurysmatic subarachnoid hemorrhage
IQR	Interquartile range
WFNS	World Federation of Neurological Surgeons
CI	Confidence interval
