# Supplementary material for: Clinical Predictors of Inpatient Mortality and Poor Postoperative Course After aSAH Microsurgical Clipping: A 10-Year Experience from a Peruvian Tertiary Care Center
Source: J Clin Med. 2025 Jul 7;14(13):4799. doi: 10.3390/jcm14134799 (PMC12250825; doi:10.3390/jcm14134799)
Supplement: Supplementary file 1 [file jcm-14-04799-s001.zip › jcm-3546275-supplementary.pdf]

Clinical predictors of inpatient mortality and postoperative complications after aneurysmatic SAH microsurgical clipping: Experience in a low-middle income country

Supplementary Material

Table of Contents Page

Supplemental Table S1 ……………………………..………………..2
Supplemental Table S2 ……………………………………..……….3
Supplemental Table S3 ………………………………..……………4
Supplemental Table S4 ……………………………………..…………6
Supplemental Table S5 ……………………………………..…………7
Supplemental Table S6 ……………………………………..…………9
Supplemental Table S7 ……………………………………..………..11
Supplemental Table S8 ……………………………………..………..12
Supplemental Table S9 ……………………………………..………..13

Table S1. STROBE Statement—Checklist of items that should be included in reports of cohort studies

	Item No	Recommendation	Page No
**Title and abstract**	1	(*a*) Indicate the study's design with a commonly used term in the title or the abstract<br>(*b*) Provide in the abstract an informative and balanced summary of what was done and what was found	1
**Introduction**			
Background/rationale	2	Explain the scientific background and rationale for the investigation being reported	2
Objectives	3	State specific objectives, including any prespecified hypotheses	2
**Methods**			
Study design	4	Present key elements of study design early in the paper	2
Setting	5	Describe the setting, locations, and relevant dates, including periods of recruitment, exposure, follow-up, and data collection	2
Participants	6	(*a*) Give the eligibility criteria, and the sources and methods of selection of participants. Describe methods of follow-up<br>(*b*) For matched studies, give matching criteria and number of exposed and unexposed	2
Variables	7	Clearly define all outcomes, exposures, predictors, potential confounders, and effect modifiers. Give diagnostic criteria, if applicable	3
Data sources/ measurement	8*	For each variable of interest, give sources of data and details of methods of assessment (measurement). Describe comparability of assessment methods if there is more than one group	3
Bias	9	Describe any efforts to address potential sources of bias	3
Study size	10	Explain how the study size was arrived at	3
Quantitative variables	11	Explain how quantitative variables were handled in the analyses. If applicable, describe which groupings were chosen and why	3
Statistical methods	12	(*a*) Describe all statistical methods, including those used to control for confounding<br>(*b*) Describe any methods used to examine subgroups and interactions<br>(*c*) Explain how missing data were addressed<br>(*d*) If applicable, explain how loss to follow-up was addressed<br>(*e*) Describe any sensitivity analyses	3
**Results**			
Participants	13*	(a) Report numbers of individuals at each stage of study—eg numbers potentially eligible, examined for eligibility, confirmed eligible, included in the study, completing follow-up, and analysed<br>(b) Give reasons for non-participation at each stage<br>(c) Consider use of a flow diagram	3
Descriptive data	14*	(a) Give characteristics of study participants (eg demographic, clinical, social) and information on exposures and potential confounders<br>(b) Indicate number of participants with missing data for each variable of interest<br>(c) Summarise follow-up time (eg, average and total amount)	4
Outcome data	15*	Report numbers of outcome events or summary measures over time	4

Main results	16	(*a*) Give unadjusted estimates and, if applicable, confounder-adjusted estimates and their precision (eg, 95% confidence interval). Make clear which confounders were adjusted for and why they were included<br>(*b*) Report category boundaries when continuous variables were categorized<br>(*c*) If relevant, consider translating estimates of relative risk into absolute risk for a meaningful time period	4
Other analyses	17	Report other analyses done—eg analyses of subgroups and interactions, and sensitivity analyses	5
**Discussion**			
Key results	18	Summarise key results with reference to study objectives	16
Limitations	19	Discuss limitations of the study, taking into account sources of potential bias or imprecision. Discuss both direction and magnitude of any potential bias	17
Interpretation	20	Give a cautious overall interpretation of results considering objectives, limitations, multiplicity of analyses, results from similar studies, and other relevant evidence	17
Generalisability	21	Discuss the generalisability (external validity) of the study results	17
**Other information**			
Funding	22	Give the source of funding and the role of the funders for the present study and, if applicable, for the original study on which the present article is based	18

*Give information separately for exposed and unexposed groups.

Note: An Explanation and Elaboration article discusses each checklist item and gives methodological background and published examples of transparent reporting. The STROBE checklist is best used in conjunction with this article (freely available on the Web sites of PLoS Medicine at http:/www.plosmedicine.org/, Annals of Internal Medicine at http:/www.annals.org/, and Epidemiology at http:/www.epidem.com/). Information on the STROBE Initiative is available at http:/www.strobe-statement.org.

Table S2. Clinical characteristics of patients operated on for aneurysmal subarachnoid haemorrhage seen in the neurosurgery service of the Hospital Nacional Arzobispo Loayza during the period 2010 - 2019.

Characteristics	N (%)
**Arterial hypertension**	
No	96 (57.5)
Yes	71 (42.5)
**Diabetes mellitus**	
No	152 (91.0)
Yes	15 (9.0)
**Obesity**	
No	162 (97.0)
Yes	5 (3.0)
**Rheumatoid arthritis**	
No	164 (98.2)
Yes	3 (1.8)
**Drug dependence**	
No	164 (98.2)
Yes	3 (1.8)
**Glasgow on admission**	

6	4 (1.9)
7	3 (1.4)
8	3 (1.4)
9	6 (2.9)
10	3 (1.4)
11	6 (2.9)
12	21 (10.0)
13	36 (17.1)
14	125 (59.5)
15	
**WFNS clinical scale at admission**	
1	123 (58.6)
2	42 (20.0)
3	20 (9.5)
4	21 (10.0)
5	4 (1.9)

WFNS: World Federation of Neurological Surgeons

Table S3. Perioperative complications of patients operated on for aneurysmal subarachnoid hemorrhage at the neurosurgery service of the Hospital Nacional Arzobispo Loayza during the period 2010 - 2019.

Complications	N (%)
**Reported intraoperative neurosurgical complications**	
None	143 (68.7)
Intraoperative rupture	52 (25.0)
Cranial nerve injury	1 (0.5)
Aneurysm base tear	2 (1.0)
Severe cerebral edema	2 (1.0)
Aneurysm blister	1 (0.5)
Intraoperative bleeding	2 (1.0)
Rebleeding in waiting time	15 (7.1)
Intraoperative Rupture + Cranial Nerve Injury	1 (0.5)
Intraoperative rupture + aneurysm base rupture + aneurysm base tear	2 (1.0)
Intraoperative rupture + Severe cerebral edema	2 (1.0)
**Reported Intraoperative Neurosurgical Complications**	
None	143 (68.7)
Intraoperative rupture	54 (26.0)
Cranial nerve injury	1 (0.5)
Aneurysm base tear	2 (1.0)
Severe cerebral edema	2 (1.0)
Aneurysm blister	1 (0.5)
Intraoperative rupture + cranial nerve injury	1 (0.5)
Intraoperative rupture + Aneurysm base rupture + Aneurysm base tear	2 (1.0)
Intraoperative rupture + Severe cerebral edema	2 (1.0)
**Reported Preoperative Neurosurgical Complications**	

None	155 (73.8)
Cerebral infarction	5 (2.4)
Hydrocephalus	4 (1.9)
Vasospasm	26 (12.38)
Other	5 (2.4)
Cerebral infarction + hydrocephalus	2 (0.9)
Cerebral infarction + vasospasm	3 (1.4)
Cerebral infarction + other	1 (0.5)
Hydrocephalus + vasospasm	2 (0.9)
Hydrocephalus + other	1 (0.5)
Cerebral infarction + Hydrocephalus + vasospasm	2 (0.9)
Cerebral infarction + vasospasm + others	1 (0.5)
Hydrocephalus + vasospasm + others	1 (0.5)
**Reported postoperative clinical complications**	
None	127 (62.6)
Pneumonia	11 (5.4)
Urinary tract infection	11 (5.4)
Operative site infection	1 (0.5)
Meningitis, ventriculitis, cerebritis	3 (1.5)
Sepsis/shock	9 (4.4)
Other	10 (4.8)
Pneumonia + urinary tract infection	2 (1.0)
Pneumonia + surgical site infection	1 (0.5)
Pneumonia + meningitis, ventriculitis, cerebrilitis	2 (1.0)
Pneumonia + sepsis/shock	8 (4.0)
Pneumonia + diabetes insipidus	1 (0.5)
Pneumonia + others	3 (1.5)
Urinary tract infection + sepsis/shock	1 (0.5)
Urinary tract infection + other	3 (1.5)
Sepsis/shock + other	3 (1.5)
Pneumonia + urinary tract infection	3 (1.5)
Pneumonia + urinary tract infection + sepsis Shock	1 (0.5)
Pneumonia + meningitis, ventriculitis, cerebrilitis + Other	1 (0.5)
Urinary tract infection + diabetes	1 (0.5)
Pneumonia + urinary tract infection + Sepsis/Shock + Diabetes insipidus + Other	1 (0.5)
**Reported postoperative clinical complications**	
No complications	127 (62.6)
Nervous system complications	8 (3.9)
Respiratory, thoracic and mediastinal complications	20 (9.8)
Immune system complications	23 (11.3)
Renal and urinary complications	15 (7.4)
Other complications	10 (4.9)

Table S4. Discharge and follow-up status of patients operated on for aneurysmal subarachnoid hemorrhage at the neurosurgery service of the Hospital Nacional Arzobispo Loayza during the period 2010 - 2019.

Characteristics	N (%)
**Glasgow at discharge**	
7	1 (0.5)
9	2 (1.1)
10	5 (2.7)
11	12 (6.4)
12	8 (4.3)
13	10 (5.3)
14	34 (18.2)
15	115 (61.5)
**Glasgow at discharge categorized**	
Mild (15-13)	159 (85.0)
Moderate (12-9)	25 (13.4)
Severe (<9)	3 (1.6)
**Modified Rankin Functional Scale**	
No symptoms	3 (1.4)
No significant disability	75 (35.7)
Mild disability	40 (19.0)
Moderate disability	36 (17.1)
Moderately severe disability	28 (13.3)
Severe disability	4 (1.9)
Death	24 (11.4)
**mRS at 6 months**	
1	12 (48.0%)
2	5 (20.0%)
3	3 (12.0%)
4	3 (12.0%)
5	2 (8.0%)
**mRS at year**	
1	12 (63.2%)
2	1 (5.3%)
3	3 (15.8%)
4	3 (15.8%)

† Some values may not add up to 212 due to missing data.

Table S5. Characteristics of patients operated on for aneurysmal subarachnoid hemorrhage in the neurosurgery service of the Hospital Nacional Arzobispo Loayza during the period 2010 - 2019, according to Glasgow Scale at discharge.

Characteristics	Glasgow at Discharge		p
	Mild (n=159), n (%)	Moderate to Severe (n=28), n (%)	
**Sex**			0.713 [a]
Female	51 (86.4)	36 (13.6)	
Male	108 (84.3)	72 (15.6)	
**Age**			**0.021** [a]
≤65 years old	140 (87.5)	20 (12.5)	
>65 years old	19 (70.4)	8 (29.6)	
**History of any chronic disease**			0.424 [a]
None	59 (85.5)	10 (14.5)	
At least 1	62 (80.5)	15 (19.5)	
**Family history of aneurysm**			1.000 [b]
No	119 (82.6)	25 (17.4)	
Yes	1 (100.0)	0 (0.0)	
**Report of headache in anamnesis**			1.000 [b]
No	2 (100.0)	0 (0.0)	
Yes	157 (84.9)	28 (15.1)	
**Time from stroke to operation (in days), median (IQR)**	2 (6.5)	2 (5.6)	0.710 [c]
**Time from stroke to operation (in days) categorized as follows**			0.648 [a]
≤ 3 days	33 (89.2)	4 (10.8)	
4 - 10 days	51 (82.3)	11 (17.7)	
> 10 days	69 (89.1)	13 (15.8)	
**Glasgow on admission categorized**			**<0.001** [b]
Mild (15-13)	149 (88.6)	19 (11.4)	
Moderate (12-9)	11 (64.7)	6 (35.3)	
Severe (<9)	0 (0.0)	3 (100.0)	
**WFNS clinical scale on admission**			**0.001** [a]
Mild (1 to 3)	149 (87.6)	21 (12.3)	
Severe (4 to 5)	10 (58.8)	7 (41.2)	
**Fisher tomographic scale on admission**			0.577 [a]
I - No evidence of bleeding in cisterns or ventricles.	9 (100.0)	0 (0.0)	
II - Thin diffuse blood, with a layer < 1 mm in cisterns measured vertically	16 (84.2)	2.8 (15.8)	

III - Thick cisternal clot, >1 mm in cisterns measured vertically	89 (83.2)	18 (16.8)	
IV - Intraparenchymal hematoma, intraventricular hemorrhage, +/- diffuse bleeding	45 (86.5)	7 (13.5)	
**Time from stroke to operation (in days) (median; RIC)**	9 (12.0)	8 (13.0)	0.490 [c]
**Number of diagnosed aneurysms**			0.077 [a]
1	133 (86.9)	20 (13.1)	
2	23 (79.3)	6 (20.7)	
3	1 (33.3)	2 (66.7)	
4	2 (100.0)	0 (0.0)	
**Approach**			0.823 [b]
Minipterional	136 (84.5)	25 (15.5)	
Supraorbital lateral	8 (80.0)	2 (20.0)	
Frontal	9 (90.0)	1 (10.0)	
Decompressive hemicraniectomy	6 (100.0)	0 (0.0)	
**Requirement for transient clipping in parenteral artery**			0.116 [a]
No	88 (88.9)	11 (11.1)	
Yes	71 (80.7)	17 (19.3)	
**Number of aneurysms clipping**			0.200 [b]
1	97 (86.6)	15 (13.4)	
2	13 (72.2)	5 (27.8)	
3	3 (100.0)	0 (0.0)	
4	-	-	
**Reported intraoperative neurosurgical complications**			0.068 [a]
None	117 (88.0)	16 (12.0)	
1 intraoperative complication	41 (77.4)	12 (22.6)	
**Reported postoperative neurosurgical complications**			**0.033 [b]**
None	130 (89.0)	16 (11.0)	
1 postoperative complication	25 (75.8)	8 (24.2)	
2 or more postoperative complications	4 (66.7)	2 (33.3)	
**Reported postoperative neurosurgical complications**			**0.018 [b]**
No complications	130 (89.0)	16 (11.0)	
At least cerebral infarction and/or vasospasm	26 (78.8)	7 (21.2)	
Other complications	3 (50.0)	3 (50.0)	
**Reported postoperative clinical complications**			**0.001 [a]**
None	109 (92.4)	9 (7.6)	
1 postoperative complication	30 (81.1)	7 (18.9)	

2 or more postoperative complications	16 (64.0)	9 (36.0)

Statistical tests: a Pearson's chi2; b Fisher's exact test; c Mann-Whitney U test. p values < 0.05 are in bold

Table S6. Poisson regression analysis predicting Glasgow Scale moderate/severe at discharge

Variables	Glasgow at discharge (Moderate to Severe)					
	Crude model			Adjusted Model*		
	RR	95% CI	p	RR	95% CI	p
**Age**						
≤65 years old		Ref.			Ref.	
>65 years old	2.37	1.16 - 4.84	**0.018**	1.84	0.87 - 3.89	0.108
**Glasgow on admission categorized**						
Mild (15-13)		Ref.			Ref.	
Moderate (12-9)	3.10	1.43 - 6.71	**0.004**	14.64	4.28 - 50.09	**<0.001**
Severe (<9)	8.79	5.75 - 13.44	**<0.001**	60.07	11.96 - 301.62	**<0.001**
**WFNS clinical scale on admission**						
Mild (1 to 3)		Ref.			Ref.	
Severe (4 to 5)	3.33	1.66 - 6.69	**0.001**	0.15	0.04 - 0.54	**0.004**
**Reported intraoperative neurosurgical complications**						
None		Ref.			Ref.	
1 postoperative complication	1.87	0.93 - 3.74	0.079	2.93	1.23 - 7.01	**0.016**
2 or more postoperative complications	2.08	0.36 - 12.12	0.416	3.43	0.67 - 17.61	0.141
**Reported postoperative neurosurgical complications**						
No complications		Ref.			Ref.	
At least cerebral infarction and/or vasospasm	1.94	0.86 - 4.33	0.108	0.99	0.41 - 2.35	0.977
Other complications	4.56	1.81 - 11.52	**0.001**	8.29	2.60 – 26.44	**<0.001**
**Reported postoperative clinical complications**						
None		Ref.			Ref.	

1 postoperative complication	2.48	0.99 - 6.22	0.053	1.42	0.62 - 3.26	0.402
2 or more postoperative complications	4.72	2.08 - 10.71	**<0.001**	4.04	1.67 - 9.79	**0.002**

RR: Risk Ratio; CI: Confidence Interval; Statistical test: Generalized linear model [family(poisson) link(log)] robust. p values < 0.05 are in bold

* Adjusted for all variables from table except Requirement for transient clipping in parenteral artery and reported intraoperative neurosurgical complications

Table S7. Sensitivity analysis for mortality risk in patients operated on for aneurysmal subarachnoid hemorrhage at the neurosurgery service of the Hospital Nacional Arzobispo Loayza during the period 2010 - 2019.

Sensitivity analysis	Variables		No. of cases/No. at risk	RR (95% CI)	p
**Sensitivity analysis 1**					
**Excluded >65 years old**	**Severe WFNS clinical scale on admission**		5/15	2.59 (0.89 - 7.55)	0.082
	**Reported intraoperative neurosurgical complications**	**1 postoperative complication**	9/35	1.91 (0.68 - 5.32)	0.219
		**2 or more postoperative complications**	6/10	0.91 (0.16 - 5.03)	0.915
	**Reported postoperative neurosurgical complications**	**At least cerebral infarction and/or vasospasm**	11/38	7.20 (1.99 - 26.00)	**0.003**
		**Other complications**	4/7	14.91 (3.71 – 59.94)	**<0.001**
	**Reported postoperative clinical complications**	**1 postoperative complication**	7/34	1.21 (0.49 - 3.01)	0.674
		**2 or more postoperative complications**	3/23	1.18 (0.28 - 5.00)	0.819
**Sensitivity analysis 2**					
**Excluded intraoperative complications**	**Age**	**>65 years old**	3/22	0.73 (0.17 - 3.21)	0.680
	**WFNS clinical scale on admission**		4/16	7.64 (1.78 - 32.83)	**0.006**
	**Reported postoperative neurosurgical complications**	**At least cerebral infarction and/or vasospasm**	5/26	5.63 (1.33 - 23.95)	**0.019**

	**Other complications**		10.37 (1.73 – 62.02)	**0.010**
**Reported postoperative clinical complications**	**1 postoperative complication**	5/23	2.99 (0.85 - 10.55)	0.089
	**2 or more postoperative complications**	2/18	0.67 (1.00 - 5.25)	0.703

RR: Risk Ratio; CI: Confidence Interval; Statistical test: Generalized linear model [family(poisson) link(log)] robust. p values < 0.05 are in bold

Table S8. Sensitivity analysis for mRS (Disability) risk in patients operated on for aneurysmal subarachnoid hemorrhage at the neurosurgery service of the Hospital Nacional Arzobispo Loayza during the period 2010 - 2019.

Sensitivity analysis	Variables		No. of cases/No. at risk	RR (95% CI)	p
**Sensitivity analysis 1**					
**Excluded >65 years old**	**Severe WFNS clinical scale on admission**		10/15	1.80 (0.91 - 3.57)	0.090
	**Reported intraoperative neurosurgical complications**	**1 postoperative complication**	9/50	1.65 (0.97 - 2.81)	0.066
		**2 or more postoperative complications**	1/5	1.67 (0.49 - 5.71)	0.411
	**Reported postoperative neurosurgical complications**	**At least cerebral infarction and/or vasospasm**	20/38	3.22 (1.78 - 5.83)	**<0.001**
		**Other complications**	6/7	6.31 (2.56 – 15.53)	**<0.001**
	**Reported postoperative clinical complications**	**1 postoperative complication**	7/34	1.74 (0.90 - 3.33)	0.098
		**2 or more postoperative complications**	3/23	3.60 (1.97 - 6.57)	**<0.001**
**Sensitivity analysis 2**					
**Excluded intraoperative complications**	**Age**	**>65 years old**	10/22	1.50 (0.80 - 2.80)	0.208
	**WFNS clinical scale on admission**		10/16	2.28 (1.14 - 4.57)	**0.020**
	**Reported postoperative neurosurgical complications**	**At least cerebral infarction and/or vasospasm**	13/26	2.82 (1.36 - 3.20)	**0.002**

	**Other complications**	5/6	5.65 (2.62 - 12.19)	**<0.001**
**Reported postoperative clinical complications**	**1 postoperative complication**	12/23	3.35 (1.62 - 6.91)	**0.001**
	**2 or more postoperative complications**	9/18	3.05 (1.29 - 7.18)	**0.011**

RR: Risk Ratio; CI: Confidence Interval; Statistical test: Generalized linear model [family(poisson) link(log)] robust. p values < 0.05 are in bold
